# The variables associated with dental anxiety and their management in primary care dental clinics in Bahrain: a cross-sectional study

**DOI:** 10.1186/s12903-022-02173-7

**Published:** 2022-04-21

**Authors:** Gowri Sivaramakrishnan, Hawra Makki, Samar AlDallal, Zahra Alaswad, Eman Sultan, Sara Ahmed, Haifa AlBanna, Muneera Alsobaiei, Leena AlSalihi

**Affiliations:** 1 Dental Postgraduate Training Department, Ministry of Health, Manama, Bahrain; 2 Private Dental Practice, Manama, Bahrain

**Keywords:** Dental fear, Dental phobic, Behavior management, Dental anxiety

## Abstract

**Background:**

20–80% of adults presenting to a dental clinic experience anxiety. Negative past dental experiences and environmental factors such as the waiting area of a dental clinic or sound of a drill are commonly considered triggering factors for anxiety. Anxiety management strategies are considered a part of routine dental procedure, due to increased prevalence and compromised patient care. Hence the aim of the present study is to identify the prevalence and variables associated with dental anxiety and their management in patients visiting the primary care dental clinics in Bahrain.

**Method:**

Four hundred and eighty participants were included. A 3-part questionnaire deciphered the demographic characteristics of the participants, the dental procedure undertaken, the level of anxiety, and the management strategy used by the dentist. The pre and post-treatment MDAS scores were recorded. Paired t test, ANOVA and Wilcoxon signed rank test was used to test the level of significance between the variables and the mean MDAS scores. The *p* ≤ 0.05 was considered statistically significant.

**Results:**

The prevalence of dental anxiety was 23.7% with moderate anxiety, and 11.4% with high anxiety. Females presented with a higher mean MDAS both pre and post-treatment compared with males. A statistically significant difference between the pre and post-treatment MDAS scores were observed in educated patients less than 50 years of age. Those with unpleasant previous dental experience showed statistically significant difference. Analyses of anxiety management techniques showed that single techniques worked better than combination techniques. Rest and breaks combined with any other technique of choice showed significant reduction in the MDAS scores post treatment.

**Conclusion:**

To conclude, all patients attending the dental clinic present with some level of anxiety that necessitates the dentist to use anxiety management strategies. Non-pharmacological methods that are non-invasive must be the first choice. Rests and breaks, with any technique of choice provides the best possible anxiety management. It is possible to achieve the desired anxiety reduction in single visit to complete the planned dental intervention, other than in patients who are dental phobic.

**Supplementary Information:**

The online version contains supplementary material available at 10.1186/s12903-022-02173-7.

## Background

Dentists deal with a number of anxious patients regularly that require incredible amount of time, care and skills to overcome their anxiety, and treat them successfully. Extremely anxious patients, unfortunately, are unable to cope up with urgent symptomatic or routine dental interventions. They are likely to postpone dental visits, or experience a negative dental experience, which further leads to fear of future dental treatments [1, 2]. Dental anxiety is indicative of a state of apprehension that something dreadful is going to happen in relation to dental treatment, and it is usually coupled with a sense of losing control. In contrast, dental fear is a normal emotional reaction to one or more specific threatening stimuli in the dental situation [3]. Dental phobia denotes a severe type of dental anxiety, and is characterized by marked and persistent anxiety in relation to either clearly discernible situations or objects like drilling, local anesthetic injections or to the dental setting in general [3]. The differential diagnoses of different types of dental anxiety that is mentioned above are subjective, and there is no clear demarcation available in literature. However, the triggering events in a dental clinic leads to a characteristic response pattern that primarily depends upon the level of perceived threat and the accompanying arousal of the patient’s defensive system. The reactions initially expressed as defensive mobility, later lead to defensive action. Dental phobia is shown to be commonly associated vasovagal responses such as an accelerated heart rate due to sympathetic activation, in contrast to dental anxiety or fear. The phobia of blood, injection and injuries (BII) is shown to be associated with initial tachycardia followed by bradycardia, hypotension, vertigo, nausea and death [4]. Dental practioners should be able to differentiate the different types of anxiety and be prepared to be able to manage any consequences.

Adult patients presenting to a dental clinic reported varied prevalence of dental anxiety from 15 to 80% from previous studies, due to the differences in the scales used to measure anxiety [5–8]. A number of factors might play a vital role in the development of dental anxiety. These include negative past dental experiences, other peoples’ experience and views, related posts on social media, pain or any other clinical symptom, personality of the patient, and environmental factors such as the waiting area of a dental clinic, sound of a drill or the smell of chemicals [5–9].

The assessment of dental anxiety is subjective with the use of interviews or self-reporting questionnaires. The objective methods measure the blood pressure, pulse rate, pulse oximetry, finger temperature, and galvanic skin response [10]. It is to be noted that treatment decisions are not completely dependent upon the anxiety scores. Subjective methods are simple to use. A baseline data on the overall dental fear is obtained in order to modify the planned treatment decisions accordingly. In contrast, the objective methods are time consuming and may involve sample collection, which may increase the existing level of anxiety [10]. Perhaps the two most frequently used adult questionnaire methods are the Dental Anxiety Scale (DAS) and the Modified Dental Anxiety Scale (MDAS). The original DAS is a 4-item questionnaire, and patients are rated based on their anxiety as they imagine approaching four dental situations: on day prior to the dental appointment, sitting in the waiting area, in the dental chair during teeth drilling, and when the teeth are cleaned. The psychometrics and content validity of DAS was further modified by adding an item on receiving dental injections. This was renamed as the Modified Dental Anxiety Scale (MDAS) [11, 12]. The MDAS has been found to be reliable and valid in several samples from England, Scotland, Wales, Ireland, Finland, Dubai, Brazil, and Turkey. MDAS was used in this study because this is already validated in the Arab population [13].

Management of dental anxiety depends on the degree of the patient’s anxiety, clinician’s skills and expertise, and the clinical situation [14, 15]. Non-pharmacological strategies such as distraction, positive reinforcement, control, systematic desensitization, rests and breaks and shortening the length of the appointment are being routinely used in the primary care dental clinics in Bahrain to manage anxious patients. Identifying dental anxiety a priori can make it possible for dentists to predict patient behavior and be better prepared with measures that will help the patient lessen their anxiety. Considering the increasing number of patients that are presenting with dental anxiety in our clinics and the related consequences in management, the aim of the present study is to identify the prevalence and various factors that are associated with dental anxiety, and their management, in patients visiting the primary care dental clinics in Bahrain.

## Method

### Study design and setting

Adult participants that attended 15 primary care dental clinics at the Ministry of Health in Bahrain were included. Participants were included irrespective of the chief complaint and the dental procedure performed. The sampling technique was a systematic random sample (every third patient who walked into the clinic was included). Fifteen general dental practitioners with no specialty training, or no special interest in anxiety management, were also included based on convenience sampling. The data was collected over a four-month period. The Primary Care Ethics committee of the Ministry of Health, Bahrain, approved the study protocol. The present study is presented according to STROBE guidelines [16]. The STROBE checklist is provided as Additional file 1: Table S1.

### Participants

Four hundred and eighty adult participants greater than 12 years of age were included. All study participants signed a written informed consent prepared according to the Declaration of Helsinki. Patients with any psychiatric disorders, on anti-anxiety medications, and did not provide a written informed consent were excluded from the study.

### Study measurement

MDAS: The MDAS is a 5-item scale used to assess fear of dental procedures before and after dental treatment, waiting for treatment, drilling, cleaning, and local anesthetic injections. Each item scored as follows 1 = not anxious; 2 = slightly anxious; 3 = moderately anxious; 4 = highly anxious; 5 = extremely high anxiety. These descriptors were explained to the study participant to clearly decipher their level of anxiety for each item of MDAS. The total score range is between 5 and 25. The total MDAS cut off points that were used to categorize patients were 5- no anxiety; 6–7 = extremely low; 8–10 = low anxiety; 11–15 = moderate anxiety; 16–19 = high anxiety; above 19 = dental phobic. In the present study pre and post-treatment MDAS was measured to assess the level of anxiety before, and following the use of anxiety management techniques, similar to previous studies [8, 17]. This is a modified version of the original MDAS described by Humphris et al*.* that we use in our dental clinics [11].

### Variables and data sources

A battery of questions was divided into 3 parts to decipher the demographic characteristics, previous dentist experience, method used for anxiety management and current dental procedure and experience. The questionnaire was administered to both the patient and the dentist. The pre-treatment MDAS was recorded in the waiting area of the dental clinic. The post-treatment MDAS was taken after the dental procedure using any anxiety management technique that is previously mentioned.

### Statistical method

Descriptive statistics was used to analyze the demographic data. Considering that, at least 3000 adult patients will visit the included dental clinics during the data collection period, at 95% confidence interval and 5% margin of error, a minimum sample size of 341 is required. Since we had more adult patients attending our clinics during the data collection period, a final sample of 480 patients were included. Cronbach’s alpha was used to check the reliability of the MDAS scale in the present sample. The mean total MDAS scores was calculated for all the variables studied. Kolmogrov Simirnov test and Shapiro Wilk test were used to check normality. Paired t test and Wilcoxon signed rank test was used to test the level of significance between the variables and the mean MDAS scores. ANOVA test was used to calculate the level of significance in the number of patients in various categories of MDAS. The *p* ≤ 0.05 was considered statistically significant. Spearman rank correlation was used to assess the strength of association between pre-treatment and post-treatment MDAS scores. All statistical tests were performed using Graph Pad Instat version 3.1 software.

## Results

### Descriptive data and mean MDAS of study participants

Three hundred and one females and 179 male patients participated in the study. The mean(SD) age of study participants was 32.66 (13.17). The mean (SD) total pre-treatment MDAS score was 10.06 (4.44). Cronbach’s alpha for the current sample was 0.77 indicating that the data from the sample is reliable. The anxiety management techniques that were used by the general dental practioners include distraction, positive reinforcement, control, systematic desensitization, rest and breaks, and short appointments. These techniques were used alone or in combination. All patients experienced some level of dental anxiety or phobia. The minimum pre-treatment total MDAS score was six and the maximum was 25. None of the participants recorded a total score of 5 indicating that none of the patients presented with no anxiety. This confirms that a minimum level of anxiety is always present in a dental clinic. 18.3% (n = 88) presented with extremely low anxiety, 41.8% (n = 201) patients with low anxiety, and 23.7% (n = 114) patients were moderately anxious 11.4% (n = 55) were highly anxious and 4.58% (n = 22) were dental phobics, according to the total MDAS score categorization (Table 1). 21 patients mentioned that this was their first dental visit. The mean pre-treatment MDAS was greatest in patients that visited the dental clinic for the first time and in patients who had unpleasant, unhappy, unsatisfied previous dental experience.

**Table 1:** Number of patients and their anxiety level based on total MDAS scores

Total MDAS Score category	Number of patients(n = 480)		Difference in the number of patients in each category
	Pre-treatment (%)	Post treatment (%)	
5 (no anxiety)	0	0	There were no patients that belonged in this category
6–7(extremely low anxious)	88(18.3)	149(31.0)	+ 61
8–10 (low anxiety)	201(41.8)	210(43.7)	+ 9
11–15(moderate anxiety)	114(23.7)	84(17.5)	− 30
16–19(high anxiety)	55(11.4)	21(4.3)	− 34
Above 19 (dental phobic)	22(4.58)	16(3.33)	− 6
	Mean 80.00 SD 72.46 SEM 29.58	Mean 80.00 SD 84.44 SEM 34.47	*P* value* = 0.002 (ANOVA test)

SD, standard deviation; SEM, standard error of mean

*P value is significant showing significant difference between the categories

### Comparison between pre and post-treatment MDAS scores

The mean (SD) of total MDAS score post-treatment was 8.41(3.89). There was a statistically significant difference between the pre and the post-treatment total MDAS scores indicating that anxiety management techniques that were used significantly reduced the dental anxiety scores, irrespective of the variables studied. The total mean MDAS scores and the corresponding p value for each item of the MDAS scale is presented in Table 2. The Spearman’s rank correlation value is 0.7 indicating a strong correlation between the pre and the post total MDAS scores. This indicates that a higher pre-treatment MDAS would likely influence the post-treatment MDAS scores. Table 3 show mean MDAS score for all the categorized variables and mean difference. The mean post-treatment MDAS was less than the pre-treatment MDAS in all the variables studied (Table 3).

**Table 2:** Mean (SD) of total MDAS scores pre- and post-dental treatment

Items in the MDAS	MDAS pre-treatment scores			MDAS post-treatment scores			*P* value (Wilcoxon signed rank test)
	Mean (SD)	SEM	95% CI	Mean (SD)	SEM	95% CI	
Visit to a dentist tomorrow	1.74 (0.996)	0.045	1.65–1.83	1.48 (0.86)	0.039	1.4–1.56	0.00001**
Sitting in the waiting room	1.77 (1.01)	0.461	0.87–2.67	1.5 (0.87)	0.039	1.42–1.58	0.000007**
Tooth drilled	2.44 (1.26)	0.057	2.33–2.55	1.97 (1.09)	0.049	1.87–2.1	0.7865
Teeth scaled and polished	1.58 (0.97)	0.044	1.49–1.67	1.38 (0.788)	0.036	1.3–1.45	0.0003**
Local anesthesia	2.49 (1.41)	0.064	2.36–2.62	2.05 (1.23)	0.056	1.94–2.16	0.000001**
Total MDAS score	10.06 (4.44)	0.2	9.67–10.452	8.41 (3.89)	0.18	8.1–8.76	0.0001**
Spearman rank correlation							
Rho 0.78 (indicates strong correlation)							

SD, standard deviation; SEM, standard error of mean; CI, confidence intervals

**p ≤ 0.05 was considered statistically significant

**Table 3:** Variables assessed in the study

Variables	Description of variables	Number of patients(n) total sample = 480	Pre-treatment MDAS		Post treatment MDAS		Mean difference	*P* value**
			Mean (SD)	95% CI	Mean (SD)	95% CI		
Gender	Female	301	10.71 (4.4)	10.2–11.2	8.90 (3.96)	8.45–9.35	1.81	0.0001*
	Male	179	8.96 (4.31)	8.3–9.6	7.60 (3.63)	7.1–8.1	1.36	0.0014*
Age	12–30 years	239	10.17 (4.32)	9.6–10.7	8.51 (3.8)	8.03–8.99	1.66	0.001*
	31–50 years	179	10.26 (4.7)	9.6–10.9	8.54 (4.24)	7.9–9.16	1.72	0.0003*
	51 and above	62	9.70 (5.11)	8.4–11	8.25 (4.36)	7.2–9.3	1.45	0.091
Education level	Educated (any level)	464	10.10 (4.46)	9.7–10.5	8.42 (3.19)	8.1–8.7	1.68	0.001*
	Uneducated	16	8.82 (3.73)	7–10.7	8.12 (3.39)	6.5–9.8	0.70	0.582
Dental visit	First	21	13.23 (7.24)	10.1–16.3	10.24 (6.03)	7.7–12.8	2.99	0.153
	Visited dentist previously	459	9.91 (4.23)	9.5–10.3	8.33 (3.75)	8–8.7	1.58	0.0001*
Gathering information prior to dentist visit	yes	207	10.69 (4.88)	10–11.4	10.03 (4.26)	9.5–10.6	0.66	0.143
	No	273	9.73 (4.03)	9.3–10.2	7.95 (3.52)	7.5–8.4	1.78	0.001*
Chief complaint	Pain	386	10.20 (4.57)	9.7–10.7	8.5 (4.04)	8.1–8.9	1.70	0.001*
	To Clean teeth	96	8.92 (3.39)	8.2–9.6	7.44 (2.85)	6.9–8	1.48	0.0013*
	Discolored or misaligned teeth	65	10 (3.13)	9.2–10.8	8.62 (3.07)	7.9–9.4	1.38	0.124*
	Missing teeth	121	10.87 (4.26)	10.1–11.6	8.25 (4.4)	7.5–9.03	2.62	0.022*
	Regular check-up	64	10.65 (3.76)	9.7–11.6	8.45 (3.19)	7.7–9.2	2.20	0.005*
Dental procedure undertaken	Endodontic access	386	10.20 (4.57)	9.7–10.7	8.5 (4.04)	8.1–8.9	1.70	0.001*
	Scaling and polishing	96	8.92 (3.39)	8.2–9.6	7.44 (2.85)	6.9–8	1.48	0.0013*
	Restorations	72	8.25 (3.12)	7.5–9	8.23 (4.05)	7.3–9.2	0.02	0.973
	Extractions	17	10.63 (4.57)	8.5–12.8	7.23 (4.87)	4.9–9.6	3.40	0.043*
Previous dental experience with rating	Extremely unpleasant with pain (5)	36	12.65 (4.82)	11.1–14.2	9.97 (4.55)	8.5–11.5	2.68	0.017*
	Not satisfying and anxious (4)	80	11.97 (3.62)	11.2–12.8	9.39 (3.14)	8.7–10.1	2.58	0.001*
	Fearful and unhappy (3)	32	12.59 (5.39)	10.7–14.5	10 (5.32)	8.2–11.8	2.59	0.049*
	Unpleasant but very slight discomfort (2)	47	11.44 (4.66)	10.1–12.8	9.55 (4.72)	8.2–10.9	1.89	0.043*
	Comfortable (1)	218	8.83 (3.83)	8.3–9.3	7.57 (3.31)	7.1–8	1.26	0.678
	Interesting/joyful(0)	46	7.72 (3.45)	6.7–8.7	7.22 (3.08)	6.3–8.11	0.50	0.350

SD, standard deviation; SEM, standard error of mean; CI, confidence intervals

*P ≤ 0.05 was considered statistically significant

**Wilcoxon signed rank test was used for non-parametric data. For parametric data, paired t test was used

### Dental procedure and the anxiety management technique used

One hundred and eighty one patients reported that local anesthesia triggered their anxiety. Other causes that were mentioned were teeth drilling for pain (234), fear of mis-diagnosis or wrong treatment (70), sound of machines (73), long appointment time (48), X-ray (15), white coat and the sight of the dentist (10). 47 patients did not know what triggered their dental anxiety. Various anxiety management techniques such as distraction, positive reinforcement, systematic desensitization, control, rest and breaks, shortening the appointment by performing only the emergency procedure, and counselling and referral at a later date, were undertaken by the dentists. Results from the present study indicate a statistically significant difference between the pre and post treatment MDAS scores using single techniques such as distraction (*p* = 0.0416), positive reinforcement (*p* = 0.0062), control (*p* = 0.0309), systematic desensitization (*p* = 0.007) and rest and breaks (*p* = 0.0219). Combination techniques were also used, which indicate significant anxiety reduction with rest and breaks combined with any other technique mentioned in this study (Table 4).

**Table 4:** Various anxiety management techniques and total MDAS scores

Anxiety management technique used (n)	Pre-treatment MDAS		Post-treatment MDAS		Mean difference	*P* value**
	Mean (SD)	95% CI	Mean (SD)	95% CI		
*Single technique used*						
Referral to specialist, recall, counselling (n = 21)	9.52 (4.3)	7.7–11.4	8.54 (4.2)	6.7–10.3	0.98	0.457
Distraction only technique (n = 15)	10.25 (3.8)	8.3–12.2	7.69 (2.68)	6.3–9	2.56	0.0416*
Positive reinforcement only technique (n = 49)	10.12 (4.3)	8.9–11.3	8.00 (3.08)	7.1–8.8	2.12	0.0062*
Control only technique (n = 57)	10.67 (4.6)	9.5–11.9	8.91 (3.95)	7.9–9.9	1.76	0.0309*
Systematic desensitization only technique (n = 10)	11.60 (3.14)	9.6–13.5	9.30 (4.19)	6.7–11.9	2.30	0.007*
Rest and breaks during the procedure only (n = 39)	10.25 (4.09)	8.9–11.5	8.23 (3.51)	7.1–9.3	2.02	0.0219*
Short appointment and only emergency procedure undertaken (n = 7)	10.71 (5.31)	6.8–14.6	10 (5.23)	6.1–13.9	0.71	0.805
*Few combination techniques are presented below*						
Distraction, positive reinforcement, Short appointment with rest and breaks (n = 2)	12 (3.41)	7.3–16.7	11.67 (4.32)	5.7–17.7	0.33	NA
Positive reinforcement, control, systematic desensitization, rest and break, short appointment (n = 2)	9.10 (5.5)	1.5–16.7	7.60 (4.72)	1.1–14.1	1.50	NA
Positive reinforcement and control (n = 12)	9.33 (1.53)	8.5–10.2	7.67 (3.06)	5.94–9.4	1.73	0.106
Distraction, positive reinforcement, systematic desensitization, control (irrespective of length of the appointment and rest and breaks)(n = 9)	10.13 (4.02)	7.5–12.8	10 (4.84)	6.8–13.2	0.13	0.951
*Significance of rest and breaks and short appointment time*						
Short appointments (irrespective of the technique used for anxiety management) (n = 31)	9.19 (3.7)	7.9–10.5	8.0 (3.89)	6.6–9.37	1.19	0.222
Rest and breaks (irrespective of the technique) (n = 149)	9.08 (3.12)	8.6–9.6	7.42 (2.5)	7–7.8	1.66	0.001*
Rest and breaks, and short appointments (irrespective of the technique) (n = 13)	10.92 (3.4)	9.1–12.8	9.08 (3.59)	7.1–11	1.84	0.192

CI, confidence intervals; NA, not assessable

*P ≤ 0.05 was considered statistically significant

**Wilcoxon signed rank test was used for non-parametric data. For parametric data, paired t test was used

### No treatment undertaken

Two patients cancelled their visit due to extreme dental phobia and disagreed to visit the dentist at a later date, 7 patients agreed to be referred to a specialist (6 patients to endodontist and 1 patient to oral surgeon) at a later date. 12 patients were counselled by the general dentist, and they agreed to visit the same dentist at a later date. No treatment was undertaken in all these patients. For those in pain, medications were prescribed. In these patients, the post-treatment MDAS was taken after counselling, discussion or prescription. Six out of the seven patients that were referred to a specialist went ahead to complete their planned treatment. Four out of the 12 patients that agreed to visit the same dentist also came back to receive their treatment.

## Discussion

The present study was aimed to identify the variables that influence dental anxiety and their management in the primary care dental clinics in Bahrain. 480 patients that experienced some form of dental anxiety measured using the MDAS scale were included. All included participants had some level or degree of dental anxiety, although majority of the patients belonged to the extremely low (18.3%) or low anxiety (41.8%) category. 23.7% had moderate anxiety and 11.4% were highly anxious. Only 4.58% presented with dental phobia. None of the patients recorded a total score of 5 (no anxiety), both pre and post treatment. Majority of the studies in the past reported similar prevalence rates of moderate to extremely high anxiety [5, 6, 18, 19], however these studies did not report the prevalence rates of extremely low or low anxiety. In the present study, 61 patients from other categories, moved to extremely low anxiety, post treatment. Patients that belong in the extremely low or low category are also anxious, and categorizing the level of anxiety will help determine the extent of anti-anxiety intervention that might be necessary. It is to be noted that 459 (95.6%) patients received the planned dental treatment using anxiety management techniques mentioned in this study, in a single visit. The 4.5% participants that did not receive any dental treatment belonged to the dental phobic category and it was not possible to reduce their anxiety in single visit using any of these techniques. Only one patient in the dental phobic category received the planned dental intervention.

Results from the present study indicate that there was significant difference between the pre and post-treatment total MDAS scores, irrespective of the gender. Particularly, significant difference was observed in four out of the five items in MDAS which included visit to a dentist tomorrow, sitting in the waiting area, teeth scaled and polished and local anesthesia. This indicates that anxiety management strategies that were used significantly influenced the level of dental anxiety. Dentists must have a thorough knowledge and must apply the commonly used non-pharmacological strategies such as distraction, rest and breaks, positive reinforcement, systematic desensitization and control [20]. This is an essential part of routine dental procedure that needs to be acquired with experience along with their clinical skills, considering the higher prevalence of dental anxiety. Majority of the previous studies reported the use of these techniques in pediatric population [20], however, the present study identifies that adult patients also experience similar range of anxiety, and the techniques mentioned above must be used to deliver effective and satisfactory treatment.

Educated adult patients less than 50 years of age, with previous unpleasant dental experience, and did not gather prior information on anxiety, showed statistically significant difference in their pre and post-treatment MDAS scores. Neither the presenting complaint nor the procedure undertaken had an influence on the difference in pre and post-treatment MDAS scores. Females presented with a higher mean MDAS both pre and post-treatment compared with males. This finding is similar to other previous studies [2, 5, 17, 21]. Those patients with unpleasant, unsatisfied and unhappy previous dental experience showed significant difference in comparison to those who experienced comfortable or interesting previous dental experience. This infers that the technique used for anxiety management produces desirable results in adult patients with bitter past dental experience, that showed greater mean pre-treatment MDAS scores.

The following anxiety management single techniques showed significant difference between the pre and post-treatment MDAS scores: Distraction, positive reinforcement, control, systematic desensitization and, rests and breaks. Analyses of combination of techniques showed that providing rest and breaks combined with any other technique of choice showed significant difference between the pre and post-treatment MDAS scores. This infers that rests and breaks help accomplish the necessary emergency intervention. It is to be noted that the sample size in combination techniques are minimum compared with the single techniques. This may have an influence on the results obtained using combination techniques. Lengthy appointments without rests and breaks have shown to produce harmful effects on the masticatory muscles and the temporo-mandibular joint [22]. This may have an influence on their behavior. Hence, our study emphasizes the need for rests and breaks in between the dental procedure. Systematic review and meta-analysis in the past on various anxiety management techniques concluded that all techniques produced similar results [20]. It is the decision of the clinician to decide on what technique or combination works better according to the clinical situation. The anxiety in adult patients can be prompted based on their opinion regarding the dentist and the dental surgery [20, 21]. Building a good rapport with the patient may help in solving the issue of dental anxiety in adults as well as in children. It also makes it easier to apply the best possible anxiety management technique of choice.

To conclude, almost all patients attending the dental clinic present with some level or degree of anxiety that necessitates the dentist to use anxiety management strategies. Non-pharmacological methods that are non-invasive must be the first choice. The dentist should decide the technique based on the clinical situation. The study is limited by the fact that only a limited number of management strategies were tested in the present study. Techniques such as tell-show-do and cognitive behavior management therapy were not included. This was done to keep the study simple. In addition, there were minimum number of participants that belong in the above 50-age category. The included patients in which the combination techniques were used was also minimum. Probably increase in the sample size in these categories may influence the results obtained.

## Supplementary Information

**Additional file 1.**  STROBE Checklist.

## Data Availability

The datasets used and/or analyzed during the current study are available from the corresponding author on reasonable request.
