# Supplementary material for: The variables associated with dental anxiety and their management in primary care dental clinics in Bahrain: a cross-sectional study
Source: BMC Oral Health. 2022 Apr 21;22:137. doi: 10.1186/s12903-022-02173-7 (PMC9026669; doi:10.1186/s12903-022-02173-7)
Supplement: Supplementary file 1 — Additional file 1. STROBE Checklist. [file 12903_2022_2173_MOESM1_ESM.pdf]

Supplementary Table 1: STROBE Statement—checklist of items

	Item No.	Recommendation	Page No.
**Title and abstract**	1	(a) Indicate the study's design with a commonly used term in the title or the abstract	Page 1,2
		(b) Provide in the abstract an informative and balanced summary of what was done and what was found	Page 3,4
**Introduction**			
Background/rationale	2	Explain the scientific background and rationale for the investigation being reported	Page 7
Objectives	3	State specific objectives, including any prespecified hypotheses	Page 7
**Methods**			
Study design	4	Present key elements of study design early in the paper	Page 8
Setting	5	Describe the setting, locations, and relevant dates, including periods of recruitment, exposure, follow-up, and data collection	Page 8,9
Participants	6	(a) Cohort study—Give the eligibility criteria, and the sources and methods of selection of participants. Describe methods of follow-up<br>Case-control study—Give the eligibility criteria, and the sources and methods of case ascertainment and control selection. Give the rationale for the choice of cases and controls<br>Cross-sectional study—Give the eligibility criteria, and the sources and methods of selection of participants	Page 8
		(b) Cohort study—For matched studies, give matching criteria and number of exposed and unexposed<br>Case-control study—For matched studies, give matching criteria and the number of controls per case	
Variables	7	Clearly define all outcomes, exposures, predictors, potential confounders, and effect modifiers. Give diagnostic criteria, if applicable	Page 9
Data sources/ measurement	8*	For each variable of interest, give sources of data and details of methods of assessment (measurement). Describe comparability of assessment methods if there is more than one group	Page 9
Bias	9	Describe any efforts to address potential sources of bias	Page 9
Study size	10	Explain how the study size was arrived at	Page 9

Continued on next page

Quantitative variables	11	Explain how quantitative variables were handled in the analyses. If applicable, describe which groupings were chosen and why	Page 9,10
Statistical methods	12	(a) Describe all statistical methods, including those used to control for confounding	Page 9
		(b) Describe any methods used to examine subgroups and interactions	NA
		(c) Explain how missing data were addressed	NA
		(d) Cohort study—If applicable, explain how loss to follow-up was addressed<br>Case-control study—If applicable, explain how matching of cases and controls was addressed<br>Cross-sectional study—If applicable, describe analytical methods taking account of sampling strategy	Page 9
		(e) Describe any sensitivity analyses	NA
**Results**			
Participants	13*	(a) Report numbers of individuals at each stage of study—eg numbers potentially eligible, examined for eligibility, confirmed eligible, included in the study, completing follow-up, and analysed	Page 11
		(b) Give reasons for non-participation at each stage	NA
		(c) Consider use of a flow diagram	NA
Descriptive data	14*	(a) Give characteristics of study participants (eg demographic, clinical, social) and information on exposures and potential confounders	Page 11
		(b) Indicate number of participants with missing data for each variable of interest	NA
		(c) Cohort study—Summarise follow-up time (eg, average and total amount)	NA
Outcome data	15*	Cohort study—Report numbers of outcome events or summary measures over time	
		Case-control study—Report numbers in each exposure category, or summary measures of exposure	
		Cross-sectional study—Report numbers of outcome events or summary measures	Page 11,12,13
Main results	16	(a) Give unadjusted estimates and, if applicable, confounder-adjusted estimates and their precision (eg, 95% confidence interval). Make clear which confounders were adjusted for and why they were included	Table 1,2,3,4
		(b) Report category boundaries when continuous variables were categorized	Table 1,2,3,4
		(c) If relevant, consider translating estimates of relative risk into absolute risk for a meaningful time period	NA

Continued on next page

Other analyses	17	Report other analyses done—eg analyses of subgroups and interactions, and sensitivity analyses	NA
**Discussion**			
Key results	18	Summarise key results with reference to study objectives	Page 14
Limitations	19	Discuss limitations of the study, taking into account sources of potential bias or imprecision. Discuss both direction and magnitude of any potential bias	Page 16
Interpretation	20	Give a cautious overall interpretation of results considering objectives, limitations, multiplicity of analyses, results from similar studies, and other relevant evidence	Page 14,15,16
Generalisability	21	Discuss the generalisability (external validity) of the study results	Page 16
**Other information**			
Funding	22	Give the source of funding and the role of the funders for the present study and, if applicable, for the original study on which the present article is based	Page 17
